# The CDC48A-PUX10 complex regulates peroxisomal protein homeostasis via ubiquitin-dependent degradation in Arabidopsis

**DOI:** 10.1093/plcell/koag202

**Published:** 2026-06-27

**Authors:** Hongwei Jing

**Affiliations:** Assistant Features Editor, The Plant Cell, American Society of Plant Biologists; Department of Horticultural Science, North Carolina State University, Raleigh, NC 27695, United States

Peroxisomes are highly dynamic, single-membrane organelles that compartmentalize diverse oxidative and metabolic reactions within eukaryotic cells. In plants, these organelles perform essential functions in fatty acid *β*-oxidation, photorespiration, reactive oxygen species (ROS) metabolism, and hormone biosynthesis, thereby playing critical roles in growth, development, signaling, and stress responses (Kao et al. 2018).

Because peroxisomes lack their own DNA, all peroxisomal proteins are nuclear-encoded and synthesized in the cytosol. These proteins are post-translationally imported into peroxisomes via conserved translocation complexes comprised of peroxin (PEX) proteins. PEX proteins also coordinate peroxisome homeostasis, including biogenesis, proliferation, and turnover (Akhter et al. 2023). Among the PEX proteins, PEX5 acts as a cytosolic receptor that recognizes and delivers cargo proteins containing a peroxisomal targeting signal type 1 (PTS1) to the peroxisomal matrix (Wang and Subramani 2017). While much is known about peroxisome biogenesis and the peroxisomal import pathway, the mechanism of peroxisome protein proteostasis that remove damaged or unnecessary peroxisomal proteins remains poorly understood. Elucidating this retrograde transport pathway is crucial for understanding how plants maintain peroxisome quality control and adapt to changing environmental conditions.

In a recent study, Jinge Li and colleagues (2026) investigated the mechanism underlying peroxisomal protein turnover. To identify proteins associated with the peroxisomal membrane, the authors employed a TurboID-based proximity labeling approach using PEX19 as the bait protein. Because PEX19 is an essential chaperone that specifically recognizes and binds peroxisomal membrane proteins (PMPs), this strategy enables the efficient labeling and identification of proteins in proximity to the peroxisomal membrane. Among the proteins identified were CDC48A, a conserved hexameric AAA+ ATPase that functions in protein quality control across eukaryotes, and its cofactor PUX10. Independent protein–protein interaction assays confirmed that PEX19 interacts with both CDC48A and PUX10. Furthermore, bimolecular fluorescence complementation (BiFC) assays demonstrated that the interaction between CDC48A and PUX10 occurs at the peroxisomal membrane.

Analysis of *cdc48a* and *pux10* mutant lines revealed a substantial accumulation of ubiquitinated proteins, indicating that CDC48A and PUX10 play critical roles in peroxisomal protein quality control. In addition, both mutants exhibited reduced peroxisome abundance, suggesting that proper proteostasis is required to maintain peroxisome homeostasis. Notably, the levels of ubiquitinated PEX5 and CAT3, a peroxisomal matrix protein, were markedly elevated in *cdc48a* and *pux10* mutants compared with the wild type. Collectively, these findings support a model in which CDC48A and PUX10 function together with PEX19 to regulate peroxisomal protein turnover, likely by facilitating the ubiquitin-dependent degradation of specific peroxisomal proteins.

Taken together, these findings by Li and colleagues (2026) demonstrate that CDC48A and its cofactor PUX10 mediate the degradation of the peroxisomal proteins PEX5 and CAT3 through the 26S proteasome pathway in Arabidopsis, thereby regulating peroxisome protein homeostasis (Fig. 1). Despite these advances, several important mechanistic questions remain unresolved. For instance, it is unclear whether additional cofactors form a complex with CDC48A to mediate peroxisomal protein degradation pathways beyond PUX10. Moreover, the identity of the E3 ligases that target peroxisomal proteins in CDC48A-dependent matrix protein degradation remains unknown, and it will be important to investigate where and how matrix proteins are ubiquitinated. Additionally, whether the CDC48A-PUX10 complex mediates the degradation of other ubiquitinated peroxisomal membrane proteins, such as PEX13 and PEX14, was not examined in this study. Another open question is whether the CDC48A complex acts cooperatively with autophagy pathways to regulate the degradation of specific peroxisomal matrix proteins, or whether it coordinately modulates CAT3 and PEX5 degradation under particular physiological conditions. Addressing these questions will deepen our mechanistic understanding of the peroxisomal protein homeostasis regulatory module.

**Figure 1:**
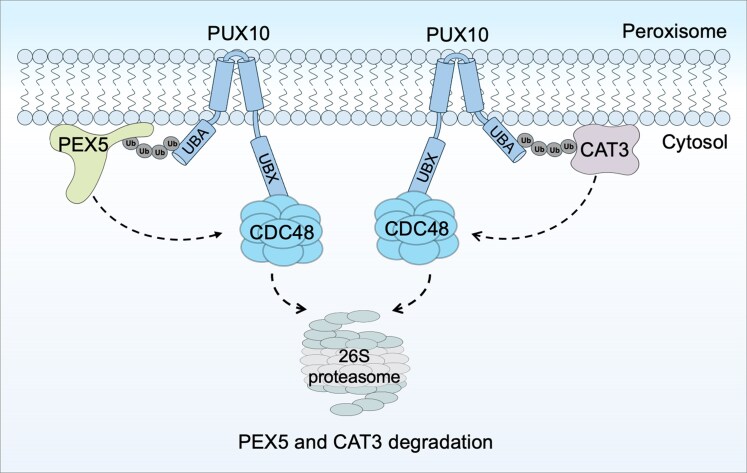
CDC48A and its cofactor PUX10 mediate ubiquitin-dependent peroxisomal protein degradation in Arabidopsis. In plant cells, the peroxisomal proteins PEX5 and CAT3 undergo polyubiquitination and are subsequently targeted for degradation. PUX10 contains an N-terminal ubiquitin-associated (UBA) domain that recognizes and binds ubiquitinated PEX5 and CAT3. PUX10 C-terminal ubiquitin regulatory X (UBX) domain serves as a cofactor for the AAA-ATPase CDC48A, recruiting CDC48A to the peroxisomal membrane. By linking CDC48A to the ubiquitinated substrates, PUX10 promotes the extraction of PEX5 and CAT3 from the peroxisomal membrane into the cytosol, where they are ultimately degraded by the 26S proteasome. Figure credit: H. Jing.

## Recent related articles in *The Plant Cell*

- Buck and coauthors (Buck et al. 2025) identified an uncharacterized Arabidopsis Pex8 protein with predicted structural similarity to yeast Pex8, revealing the conservation of peroxisomal protein import machinery across eukaryotes.
- Chen and colleagues (Chen et al. 2026) combined large-scale protein structure analysis with experimental characterization of yeast PEX8, showing that plant PEX8-like proteins functionally complement yeast pex8 mutants.
- Wang and colleagues (Wang et al. 2025) identified peroxisomal cinnamate: CoA ligases (CNLs) from Arabidopsis and showed CNL as the most functionally specific enzyme among the known enzymes of the phenylalanine ammonia lyase (PAL) pathway.
- Zhou and colleagues (Zhou et al. 2026) discovered that genetic disruption of lipases such as *SUGAR-DEPENDENT 1* (*SDP1*) in poplar increases cuticular thickness and drought resistance, suggesting that blocking peroxisomal entry redirects acyl chains toward cutin biosynthesis.

## Data Availability

No new data were generated or analysed in support of this research.

## References

[koag202-B1] Akhter D, Zhang Y, Hu JP, Pan R. 2023. Protein ubiquitination in plant peroxisomes. J Integr Plant Biol. 65:371–380. 10.1111/jipb.13346. 35975710

[koag202-B2] Buck GC, Weeks AD, Ordner NE, Bartel B. 2025. Identifying and characterizing a missing peroxin-PEX8-in Arabidopsis thaliana. Plant Cell. 37:koaf166. 10.1093/plcell/koaf166. 40577590 PMC12264594

[koag202-B3] Chen J et al 2026. Structure-guided discovery of protein functions in plants. Plant Cell. 38:koag022. 10.1093/plcell/koag022. 41662342

[koag202-B4] Kao YT, Gonzalez KL, Bartel B. 2018. Peroxisome function, biogenesis, and dynamics in plants. Plant Physiol. 176:162–177. 10.1104/pp.17.01050. 29021223 PMC5761812

[koag202-B5] Li J et al 2026. CDC48A and the UBA-domain protein, PUX10, regulate the ubiquitin-dependent degradation of peroxisomal proteins in Arabidopsis. Plant Cell. 38:koag149. 10.1093/plcell/koag149. 42203491

[koag202-B6] Wang W, Subramani S. 2017. Role of PEX5 ubiquitination in maintaining peroxisome dynamics and homeostasis. Cell Cycle. 16:2037–2045. 10.1080/15384101.2017.1376149. 28933989 PMC5731411

[koag202-B7] Wang Y et al 2025. Species- and organ-specific contribution of peroxisomal cinnamate:CoA ligases to benzoic and salicylic acid biosynthesis. Plant Cell. 37:koae329. 10.1093/plcell/koae329. PMC11708837 39692580

[koag202-B8] Zhou L et al 2026. Breakdown of lipid droplets by the triacylglycerol lipase sugar dependent 1 contributes to cuticle assembly in poplar. Plant Cell. 38:koag083. 10.1093/plcell/koag083. 41846569

